# Treatment outcome of IDH1/2 wildtype CNS WHO grade 4 glioma histologically diagnosed as WHO grade II or III astrocytomas

**DOI:** 10.1007/s11060-024-04585-7

**Published:** 2024-02-07

**Authors:** Naureen Keric, Harald Krenzlin, Darius Kalasauskas, Christian F. Freyschlag, Oliver Schnell, Martin Misch, Christian von der Brelie, Jens Gempt, Aleksandrs Krigers, Arthur Wagner, Felipa Lange, Dorothee Mielke, Clemens Sommer, Marc A. Brockmann, Bernhard Meyer, Veit Rohde, Peter Vajkoczy, Jürgen Beck, Claudius Thomé, Florian Ringel

**Affiliations:** 1 Department of Neurosurgery, University Medical Center Mainz, Johannes Gutenberg University of Mainz, Langenbeckstr. 1, 55131 Mainz, Germany; 2 Department of Neurosurgery, Medical University of Innsbruck, Innsbruck, Austria; 3 Department of Neurosurgery, Medical Center University of Freiburg, Freiburg, Germany; 4 Department of Neurosurgery, Charité University Berlin, Berlin, Germany; 5 Department of Neurosurgery, University Medical Center Göttingen, Göttingen, Germany; 6 Department of Neurosurgery, Technical University Munich, Munich, Germany; 7 Institute of Neuropathology, University Medical Center Mainz, Mainz, Germany; 8 Department of Neuroradiology, University Medical Center Mainz, Mainz, Germany

**Keywords:** IDH1/2 wildtype, WHO grade II or III astrocytomas, Glioblastoma, Extend of resection, Prognostic factors

## Abstract

**Background:**

Isocitrate dehydrogenase** (**IDH)1/2 wildtype (wt) astrocytomas formerly classified as WHO grade II or III have significantly shorter PFS and OS than IDH mutated WHO grade 2 and 3 gliomas leading to a classification as CNS WHO grade 4. It is the aim of this study to evaluate differences in the treatment-related clinical course of these tumors as they are largely unknown.

**Methods:**

Patients undergoing surgery (between 2016–2019 in six neurosurgical departments) for a histologically diagnosed WHO grade 2–3 IDH1/2-wt astrocytoma were retrospectively reviewed to assess progression free survival (PFS), overall survival (OS), and prognostic factors.

**Results:**

This multi-center study included 157 patients (mean age 58 years (20–87 years); with 36.9% females). The predominant histology was anaplastic astrocytoma WHO grade 3 (78.3%), followed by diffuse astrocytoma WHO grade 2 (21.7%). Gross total resection (GTR) was achieved in 37.6%, subtotal resection (STR) in 28.7%, and biopsy was performed in 33.8%. The median PFS (12.5 months) and OS (27.0 months) did not differ between WHO grades. Both, GTR and STR significantly increased PFS (P < 0.01) and OS (P < 0.001) compared to biopsy. Treatment according to Stupp protocol was not associated with longer OS or PFS compared to chemotherapy or radiotherapy alone. EGFR amplification (P = 0.014) and TERT-promotor mutation (P = 0.042) were associated with shortened OS. MGMT-promoter methylation had no influence on treatment response.

**Conclusions:**

WHO grade 2 and 3 IDH1/2 wt astrocytomas, treated according to the same treatment protocols, have a similar OS. Age, extent of resection, and strong EGFR expression were the most important treatment related prognostic factors.

**Supplementary Information:**

The online version contains supplementary material available at 10.1007/s11060-024-04585-7.

## Introduction

IDH wildtype (wt) astrocytomas, despite being considered as WHO grade II or III based on histologic criteria alone (e.g., no microvascular proliferation or necrosis), have poorer overall survival (OS) when compared with IDH mutated astrocytomas WHO grade II or III [1–4]. The aggressive clinical course of these tumors can be almost equal to or slightly better than IDH wt glioblastomas, classified as glioblastomas according to histologic criteria [1].

In the 2016 WHO classification of CNS tumors, molecular markers, in addition to histologic criteria, were included for the first time to type and grade gliomas [5]. Subsequently, the Consortium to Inform Molecular and Practical Approaches to CNS Tumor Taxonomy (C-IMPACT NOW initiative) increasingly precise categorized CNS tumors based on more specific molecular markers, facilitating more adequate prognostic assessments [6]. Since the publication of the third update of the Consortium to Inform Molecular and Practical Approaches to CNS Tumor Taxonomy (cIMPACT-NOW Update 3) in 2018, a subset of gliomas, which showed magnetic resonance imaging (MRI) characteristics and histological findings consistent with diffuse astrocytomas grade II and III were classified as grade 4 tumors with molecular features of glioblastoma. This classification was based on the detection of one or more of the following markers: (i) telomerase reverse transcriptase (TERT) promoter mutation, (ii) a combined complete gain of chromosome 7 and loss of chromosome 10 (+ 7/ − 10), and (iii) epidermal growth factor receptor (EGFR) amplification [1]. These tumors account for approximately 12% of all gliomas [7]. Due to the significantly more aggressive tumor behavior resembling that of glioblastoma, practitioners tended to apply high-grade glioma treatment regimens [2–4, 8–10]. According to the recent 2021 WHO classification, these tumors of histologically lower grade tumors are now being categorized as "glioblastoma IDH-wildtype (CNS WHO grade 4)" [5]. Despite this re-classification, their clinical course appears to be similar but not identical to classic glioblastomas [11]. Therefore, the postoperative treatment of diffuse IDH wt astrocytomas poses challenges and findings from retrospective studies primarily involve small cohorts and allow no firm conclusions while prospective study data are scarce. The randomized, open-label, phase 3 CATNON trial offers evidence that temozolomide adjuvant but not concurrent to a radiotherapy is associated with a survival benefit in non 1p/19q co-deleted anaplastic glioma with IDH1/2 mutation, however, not effective in IDH1/2 wildtype tumors [12, 13].

Therefore, the main objective of this study was to evaluate progression free and overall survival (PFS and OS) after surgery and adjuvant treatment of tumors formerly classified as diffuse IDH wt astrocytomas WHO grade II or III in the largest cohort so far.

## Methods

### Patient samples, study design, and outcome measures

A retrospective multi-center database analysis of diffuse IDH wt astrocytomas included surgically treated consecutive patients from six neurosurgical university departments in Germany and Austria over four years (2016–2019).

Inclusion criteria: Newly diagnosed diffuse IDH wt astrocytomas histologically graded as WHO grade II or III in patients ≥ 18 years at the time of diagnosis and at least one postoperative follow-up ≥ 3 months after surgery. Demographic and clinical data such as sex, age at surgery, tumor location, tumor size, the extent of tumor resection, neuropathological parameters, postoperative adjuvant treatment, follow-up duration, progression rates and survival were assessed.

Postoperative follow-up was performed by clinical investigation and evaluation of neuroimages either from magnetic resonance imaging (MRI) or, if not available/contraindicated, computed tomography (CT) scans. Progression/ recurrence was recorded when tumor regrowth was observed in follow-up imaging according to RANO criteria.

Pathological diagnosis was based on 2016 WHO criteria for CNS tumor classification, and the c-IMPACT NOW Update 3 [1, 7]. Tumor marker analysis was performed using established and validated methods based on the preference of the participating center.

Volumetric analysis of tumor size and extent of resection was performed on T2-weighted images, T2-weighted fluid attenuation inversion recovery images, and T1-weighted MRI images before and after applying intravenous contrast agent using a navigation planning software (iPlan 2.1, Brainlab, München, Germany). GTR was defined as resection of > 95% of the tumor. Extent of resection was assessed by two independent observers. In uncertain cases, a third observer was involved for the decision.

### Molecular analysis

Molecular analysis was performed as part of the routine diagnostic according to the current standards of diagnostics for brain tumors informed by the cIMPACT-now updates.

All hospitals used methylation-specific PCR (MSP) for MGMT-promotor methylation analysis. Negative MGMT methylation levels for qMSP were below the cut-off point of 0.35. IDH-mutation status and ATRX expression were analyzed using immune-staining. Additionally, IDH and TERT were analyzed using Sanger Sequencing of genomic DNA from formalin-fixed, paraffin-embedded samples, quantitative Real-Time PCR for EGFR amplification analysis.

### Statistical analysis

Analyses were performed using SPSS for Windows, Version 24.0. For statistical data evaluations, a descriptive analysis was conducted. Categorical data were described by absolute and relative frequency, and continuous data were described by the mean, standard error (SE), and range. PFS was defined as the time from surgery to tumor regrowth detection by imaging.

In univariate analyses, age, sex, contrast enhancement, tumor multifocality, WHO grade, molecular tumor markers (loss of nuclear ATRX, EGFR amplification, MGMT promoter methylation, and TERT promoter mutation), extent of resection, patient performance, and perioperative complications were assessed as potential risk factors for OS and PFS. A backward conditional method using all statistically significant factors from univariate regression analyses was used to select significant factors for multivariate regression analyses. A p-value < 0.05 was considered statistically significant. Adjustment for multiple testing was not performed.

## Results

### Demography

Our analysis included 157 patients. The mean age at the diagnosis was 58 years (range = 20–87 years), and 36.9% were female. The median follow-up was 12.5 months (standard deviation (SD) = 14.0, range = 0–65 months). Tumor locations were: temporal (29 patients; 18%), followed by frontal (28 patients; 18%), parietal (18 patients; 11%), insular (8 patients; 5%), posterior fossa and brainstem (7 patients; 4%), thalamic (6 patients; 4%), and occipital (1 patient; 1%). Three lobes and more were infiltrated in 33 patients (21%), and a bilobular location was found in 27 patients (17%). The median ECOG-score remained unchanged from the time of admission (1; SD = 0.90) to discharge (1; SD = 1.1). GTR (> 95%) was achieved in 59 patients (37.6%), subtotal resection in 45 patients (28.7%), and 53 patients (33.8%) underwent a biopsy only (Table 1).

**Table 1:** Baseline demographics and histopathology

	All patients
Patients, n	157
Age, mean (SD)	58.3 (13.8)
Female (%)	36.9
ECOG at admission, median (SD)	1 (0.9)
ECOG at discharge, median (SD)	1 (1.1)
Tumor localization (%)	
Unifocal	73.9
Multifocal	26.1
Extent of resection (%)	
GTR	37.6
STR	28.7
Biopsy	33.8
PFS in months, median (SE)	12.5 (1.2)
OS in months, median (SE)	27.0 (2.9)
MGMT promoter (%)	
Analyzed in total	137 (87.3)
Methylated	55 (35.0)
Unmethylated	82 (52.2)
TERT, n (%)	
Analyzed in total	40 (25.4)
Mutated	28 (17.8)
Wild-type	12 (7.6)
ATRX, n (%)	
Analyzed in total	127 (80.8)
Retained	115 (73.2)
Lost	12 (7.6)
EGFR, n (%)	
Analyzed in total	38 (24.2)
Negative	15 (9.6)
Weakly positive	3 (1.9)
Moderately positive	7 (4.5)
Strongly positive	13 (8.3)

### Survival data

Median progression free survival of the whole cohort was 12.5 ± 1.2 months, median overall survival 27.0 ± 2.9 months.

Univariate analyses of predictors of PFS and OS were performed by categorizing patients according to age, sex, ECOG status at admission and discharge, molecular markers, extent of resection, WHO grade, and adjuvant treatment. Age significantly influenced tumor progression and OS (PFS: Hazard ratio (HR) 1.02 (1.004 – 1.04) and OS: HR = 1.04 (1.02 – 1.06) for every additional year of age). Patients > 60 years had a significantly shorter PFS by univariate analysis (n = 74; HR: 1.688, 95% CI: 1.1 – 2.6, P = 0.02) when compared with patients < 60 years (n = 83).

Patients with ring-enhancing lesions had similar PFS but and OS compared with patients without contrast enhancement (HR: 2.34, 95% CI: 0.98–5.57; P = 0.056 and HR: 2.01, 95% CI: 0.59 – 6.84, P = 0.266, respectively). Functional performance was a significant influencing factor for PFS and OS (HR: 1.55, 95% CI: 1.20 – 1.99; P = 0.001 and HR: 1.55, 95% CI: 1.23 – 1.95, P < 0.001, for 1-point increase in ECOG score). Tumor multifocality was not relevant regarding PFS but was associated with a shorter OS (HR: 2.32, 95% CI: 1.40 – 3.90, P = 0.001) (Tables 1, 2).

**Table 2:** Association of patient- and tumor characteristics with progression-free and overall survival (univariate analysis)

	PFS	OS
	HR (95% CI)	HR (95% CI)
Age, years*(for every additional year)	1.02 (1.004–1.04), P = 0.014	1.04 (1.02–1.06), P < 0.001
Sex (male vs. female)	1.24 (0.80–1.91), P = 0.345	1.40 (0.82–2.40), P = 0.213
ECOG at admission* (for 1-point increase in grade)	1.55 (1.20–1.99), P = 0.001	1.55 (1.23–1.95), P < 0.001
Extent of resection		
GTR vs. biopsy*	0.28 (0.16–0.47), P < 0.001	0.15 (0.08–0.30), P < 0.001
STR vs. biopsy*	0.44 (0.25–0.74), P = 0.002	0.29 (0.16–0.54), P < 0.001
GTR vs. STR	0.64 (0.38–1.07), P = 0.088	0.53 (0.26–1.07), P = 0.077
Radiology		
Ring-enhancement vs. no	2.34 (0.98–5.57), P = 0.056	2.01 (0.59–6.84), P = 0.266
Midline shift	1.02 (0.66–1.57), P = 0.933	0.81 (0.48–1.37), P = 0.432
Multifocal tumor*	0.83 (0.51–1.36), P = 0.454	2.32 (1.40–3.90), P = 0.001
WHO Grade		
WHO grade II vs. III	0.82 (0.49–1.38), P = 0.459	0.59 (0.31–1.11), P = 0.103
Molecular markers		
MGMT methylated vs. unmethylated	0.78 (0.49–1.24), P = 0.293	0.93 (0.55–1.58), P = 0.784
ATRX lost vs. retained	0.43 (0.17–1.07), P = 0.070	0.48 (0.15–1.56), P = 0.224
EGFR expression		
No vs. strong	0.44 (0.15–1.24), P = 0.120	0.22 (0.07–0.70), P = 0.011
No-moderate vs. strong*	0.30 (0.11–0.81), P = 0.017	0.16 (0.05–0.47), P = 0.001
TERT mutation vs. wild-type*	1.65 (0.59–4.63), P = 0.34	7.85 (1.03–59.7), P = 0.047
Treatment		
Radiochemotherapy, according to Stupp vs. other treatment	0.98 (0.51–1.91), P = 0.960	0.79 (0.39–1.58), P = 0.498
Perioperative complication*	1.42 (0.75–2.69), P = 0.281	2.49 (1.39–4.47), P = 0.002

Cox regression, asterisk marks statistically significant variables.

### WHO grade and molecular markers

**Fig. 1:**
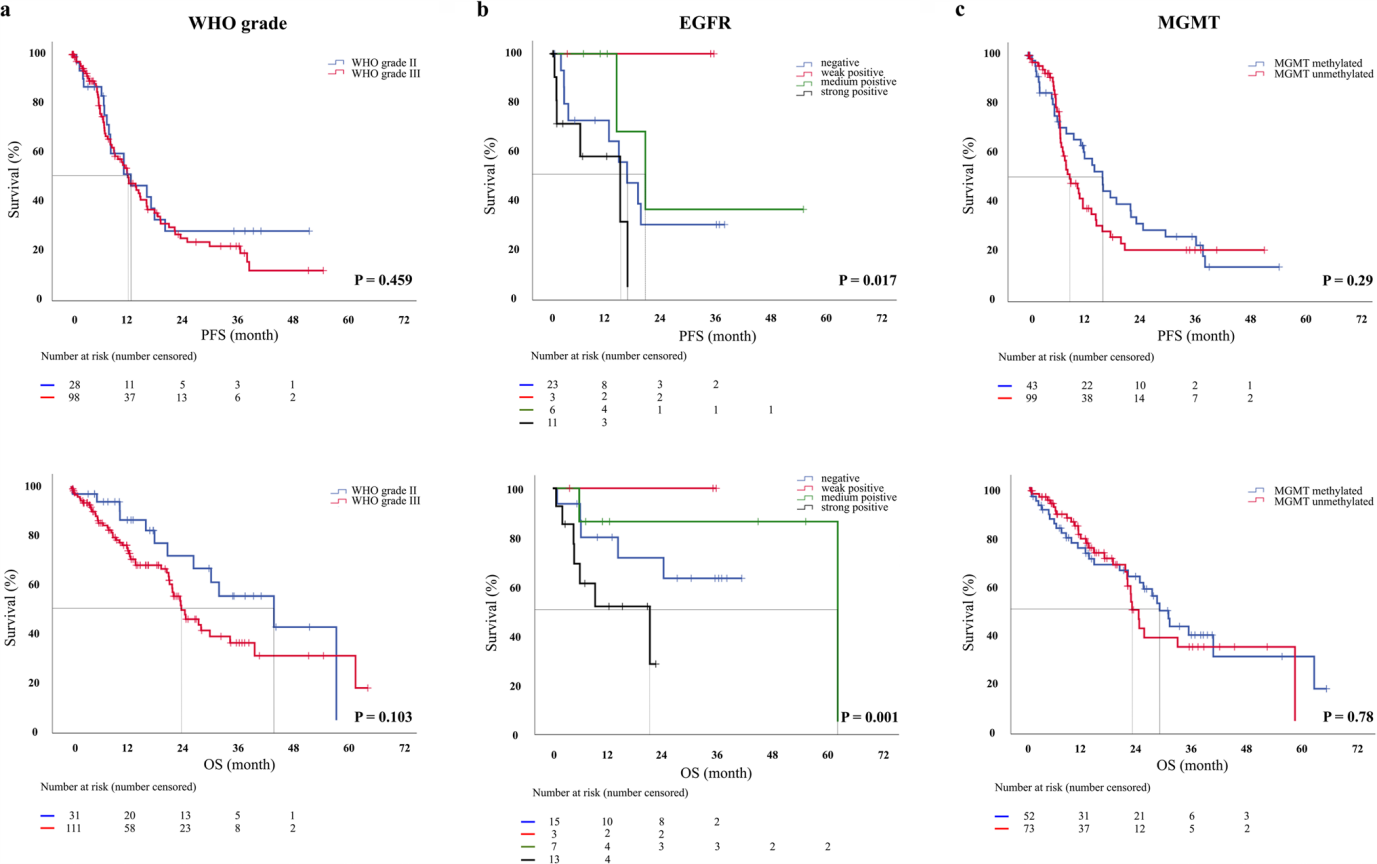
Progression-free survival and overall survival curves (a: WHO grade; b: EGFR expression; c: MGMT promotor methylation)

In 123 patients (78.3%), the predominant histology was anaplastic astrocytoma WHO grade III, followed by diffuse astrocytomas WHO grade II in 34 patients (21.7%). WHO grade was not associated with PFS or OS (HR: 0.82, 95% CI: 0.49 – 1.38, P = 0.459 and HR: 0.59, 95% CI: 0.31 – 1.11, P = 0.103, respectively) (Fig. 1a).

Not all molecular markers were assessed on a routine basis in the participating centers. In 41 patients, the TERT promoter was analyzed, with 28 (17.8%) mutated. EGFR amplification was examined in 38 patients; 15 (9.6%) were negative, 3 (1.9%) were weakly positive, 7 (4.5%) were moderately, and 13 (8.3%) were strongly positive. Nuclear ATRX loss was analyzed in 127 patients (80.8%); expression was retained in 1156 (73.2%) but lost in 12 patients (7.6%). MGMT-promoter methylation was examined in 137 patients; data were not available in 20 patients (12.7%), while 55 (35.0%) had a methylated MGMT promoter and 82 (52.2%) a non-methylated MGMT promoter (Table 1). Among all molecular markers, only strong EGFR expression was associated with a shorter PFS (HR: 3.39, 95% CI:1.24 – 9.28, P = 0.017) and OS (HR: 6.26, 95% CI:2.11 – 18.59, P = 0.001) (Fig. 1b). Patients with nuclear ATRX expression loss had a slightly longer PFS (HR: 0.43, 95% CI: 0.17 – 1.07, P = 0.07). TERT promoter mutation had no influence on PFS (P = 0.34) but was associated with shortened OS (23.5 (SE = 3.6) vs. 35.0 (SE = 3.0) months; HR: 7.84, 95% CI: 1.03 – 59.7, P = 0.047). MGMT promoter methylation did not result in significant changes of PFS (HR: 0.78, 95 CI: 0.49—1.24, P = 0.29) or OS (HR: 0.93, 95% CI: 0.55–1.58, P = 0.78). (Fig. 1c, Table 2).

### Extent of resection

**Fig. 2:**
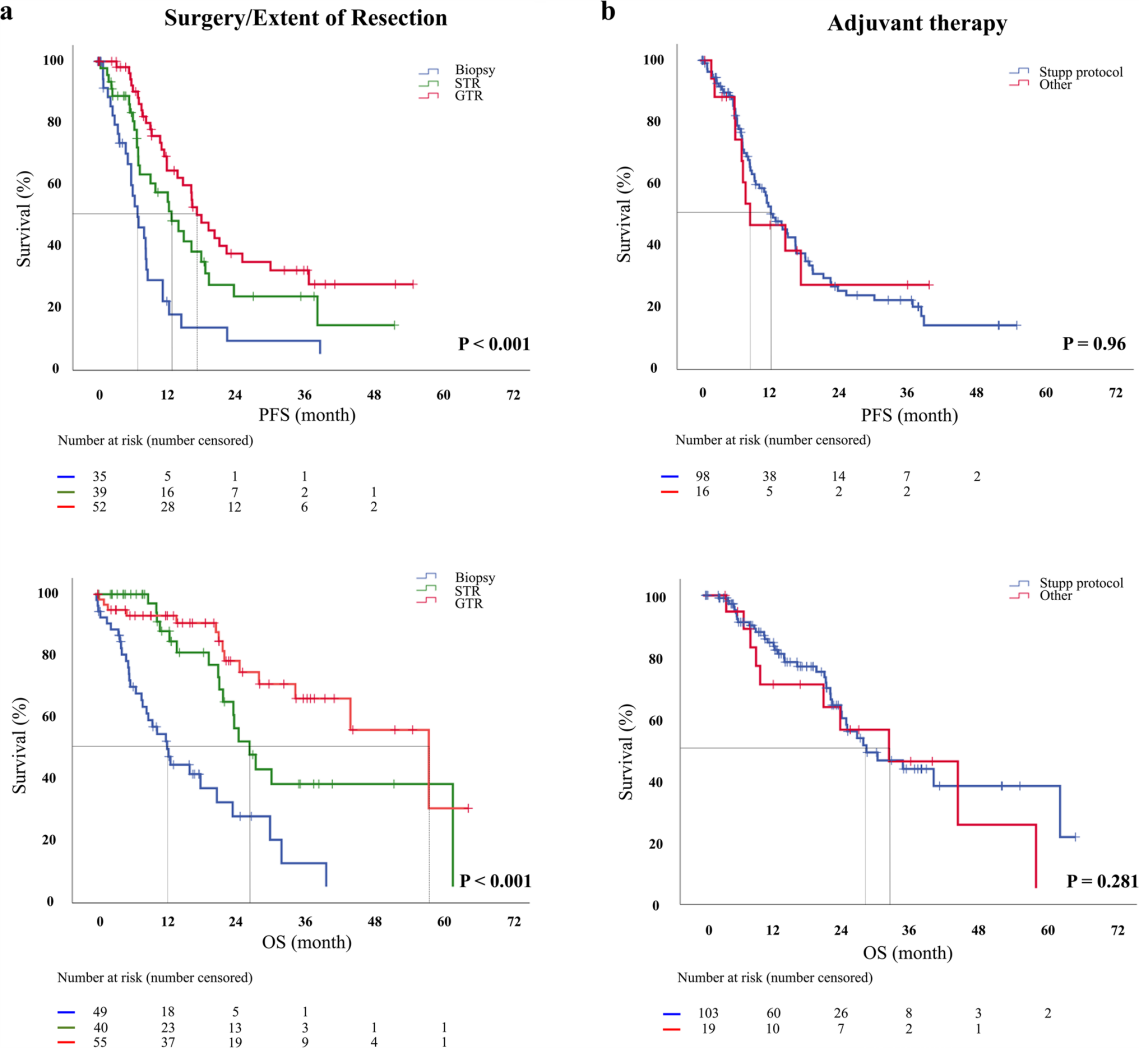
Progression-free survival and overall survival curves (a: Extent of resection (EOR); b: adjuvant treatment Stupp vs. other

The extent of tumor resection was a significant factor for progression and patient survival. Both GTR and STR prolonged PFS (HR: 0.28, 95% CI: 0.16 – 0.47, P < 0.001 and HR: 0.44, 95% CI: 0.25 – 0.74, P = 0.002, respectively) and OS (HR: 0.15, 95% CI: 0.08 – 0.30 P < 0.001 and HR: 0.29, 95% CI: 0.16–0.54, P < 0.001, respectively) when compared with biopsy alone (Fig. 2a) (Table 2). However, no significant difference was observed in survival between GTR and STR. Perioperative complications were also associated with a shorter OS (HR: 2.49, 95% CI: 1.39 – 4.47, P = 0.002).

### Non-surgical treatment data

Adjuvant treatment was performed in 132 patients (84.1%), while 111 patients received radio-chemotherapy according to the Stupp regimen (70.7%) [11], three patients (1.9%) had sequential treatment (radiotherapy followed by chemotherapy), nine patients (5.7%) had radiotherapy alone, and the same number of patients (5.7%) had chemotherapy alone [14, 15]. Treatment decisions were made at local interdisciplinary tumor conferences based on clinical status, the extent of resection, and histopathological findings, including molecular markers. Radiation treatment or chemotherapy alone with temozolomide was applied more often than concomitant therapy in patients with high ECOG and older age. The decision for radiotherapy or chemotherapy was stratified according to MGMT-promoter methylation status. Seven out of 9 patients undergoing chemotherapy alone had a methylated MGMT-promoter (the promotor methylation status was unavailable in 2 patients), while the majority (7/9) of patients undergoing radiotherapy alone had an unmethylated MGMT-promoter. The patients selected for combined radio-chemotherapy differed between 53 and 100% among the centers (Supplement Table 1). Tumor recurrence was observed in 86 (54.4%) patients. The median PFS was 12.5 months (SE = 1.2 months) and OS 27.0 months (SE = 2.9 months). PFS rate at 6 months was 84%. Treatment according to the Stupp regimen did not influence PFS when compared with other treatment regimens at different WHO grades (WHO II: P = 0.809; WHO III: P = 0.908) (Fig. 2b). When considering different WHO grades regarding OS and treatment regimens, the Stupp regimen was not associated with a longer OS in WHO grade II or III tumors either (WHO II: P = 0.495; WHO III: P = 0.221).

### Multivariate analysis

Variables associated with statistically significant differences in PFS or OS in univariate analyses (patient age, ECOG performance status, extent of resection, tumor multifocality, TERT mutation, EGFR expression, and the presence of perioperative complications) as well as several putatively clinically significant variables (WHO grade and treatment according to Stupp) were included in a multivariate survival analysis.

The extent of resection—GTR vs. biopsy (HR: 0.23, 95% CI: 0.13–0.42, P =  < 0.001), STR vs. biopsy (HR: 0.51, 95% CI: 0.29–0.90, P = 0.021), and strong EGFR expression (HR: 6.15, 95% CI: 2.15–17.60, P = 0.001) remained statistically significant factors for PFS. The 2-year PFS was 23.7% (SE = 4.4%) for the entire cohort when different WHO grades were considered: WHO II: 24.9% (SE = 9.2%), WHO III: 23.3% (SE = 5.0%) (Table 3).

**Table 3:** Association of patient- and tumor characteristics with survival (multivariate analysis)

	HR (95% CI)	P value
Progression-free survival		
Multifocality	1.74 (1.00–3.02)	0.049
Extent of resection		
Gross-total vs. biopsy	0.23 (0.13–0.42)	< 0.001
Subtotal vs. biopsy	0.51 (0.29–0.90)	0.021
Strong EGFR expression	6.15 (2.15–17.60)	0.001
Overall survival		
Extent of resection		
Gross-total vs. biopsy	0.16 (0.08–0.31)	< 0.001
Subtotal vs. biopsy	0.37 (0.19–0.71)	0.03
WHO Grade, II vs. III	0.35 (0.18–0.70)	0.003
Strong EGFR expression	7.33 (2.21–24.35)	0.001
Perioperative complication	2.00 (1.05–3.79)	0.034
TERT mutated vs. wild-type	13.58 (1.69–109.04)	0.014

Multivariate Cox Proportional Hazards Regression analysis, backward conditional selection method used, step 6 is displayed for PFS and step 4 for OS.

Similarly, OS was mainly influenced by the extent of resection—GTR vs. biopsy (HR: 0.16, 95% CI: 0.08–0.31, P =  < 0.001), STR vs. biopsy (HR: 0.37, 95% CI: 0.19–0.71, P = 0.03), the WHO grade II versus III (HR: 0.35; 95% CI:0.18–0.70, P = 0.003), strong EGFR expression (HR: 7.33, 95% CI: 2.21–24.35, P = 0.001), TERT promoter mutation (HR: 13.58, 95% CI 1.69–109.04, P = 0.014) and the perioperative complication rate (HR: 2.00, 95% CI: 1.05–3.79, P = 0.034). The 2-year OS was 57.4% (SE = 4.9%) for the entire cohort when different WHO grades were considered: WHO II: 71.9% (SE = 9.3%), WHO III: 53.1% (SE = 5.7%).

## Discussion

Diffuse IDH1/2 wt gliomas are a heterogeneous group of tumors, which in most cases will now be classified as glioblastomas, IDH-wildtype (CNS WHO grade 4) after performing the required molecular diagnostic workup according to the current WHO classification 2021 [5, 16]. Although clinicians have identified high recurrence rates, imaging features of these tumors are similar to low-grade gliomas, with histological criteria of WHO grade IV tumors such as microvascular proliferation and necrosis not detected [17–20]. In recent decades, increased knowledge of molecular profiling has provided more precise tumor information and explained more aggressive tumor behaviors. Certain molecular markers have been extensively studied in recent years; in 2018, the cIMPACT-NOW initiative implemented three of these markers- namely EGFR amplification, a combined gain of chromosome 7/ loss of chromosome 10, or TERT promoter mutation, in routine clinical diagnostics [1, 9, 10].

In this tumor group, clinicians have recorded higher recurrence rates and more aggressive growth patterns [2–4, 21, 22]. Therefore, treatment strategies have shifted towards treatment regimens for high-grade gliomas. However, the findings describing the course of this specific tumor subgroup are primarily derived from retrospective studies. Adjuvant treatment data that support treatment guidelines are simply unavailable – study cohorts are small, which hamper outcomes and conclusions [17–19]. Therefore, our remit was to assess the influence of treatment regimens by analyzing the clinical course of a large cohort of IDH1/2 wt diffuse astrocytoma patients from six neurosurgical sites treated in the 2016–2019 period. Despite the retrospective nature of our study, ours is the largest multi-center study consisting of 158 patients to analyze PFS, OS, and prognostic factors in these new glioblastomas.

The mean age of 58 years was consistent with other classical glioblastoma reports [18, 19, 23]. Age distribution of IDH1/2 wt diffuse astrocytomas have been reported to vary among different WHO grades. While patients with WHO grade II and III are younger (45 years), those with WHO grade IV tumors tend to be older (IDH1/2 wt astrocytoma with molecular features of a WHO grade IV tumor: 58 years; IDH1/2 wt glioblastomas: 55 years) [17, 24]. IDH1/2 wt astrocytomas, with TERT mutation only, have the highest age of onset (62 years) [17, 24]. The presented cohort fits into the expected age of onset while being on the upper end of age distribution. TERT mutation analysis was performed in 25.9% of all patients. Therefore, undiagnosed IDH1/2 wt astrocytomas with TERT mutation only might impact our cohort's tendency towards a higher age of onset. Tumors occurred slightly more often in males (62.7%). Traditionally, diffuse gliomas are non-sex-specific, and malignant gliomas occur more frequently in males [11, 17]. Since many of these tumors between WHO grade II-III included in our study are now considered as glioblastomas, IDH-wildtype CNS WHO grade 4, this might explain our cohort's tendency toward the male sex. ECOG performance status was univocally good and remained unaltered prior to (ECOG 1) and post-surgery (ECOG 1) in our data set. Similar good performance indices are reported in other cohorts with lower WHO grades that perform better after resection than WHO grade IV glioblastomas [17, 24]. As recommended in the most recent EANO guidelines, immunohistochemistry for mutant IDH1 R132H protein or IDH1 and IDH2 sequencing in cases with lack of IDH1 R123H immunopositivity as well as nuclear expression of ATRX should be performed routinely in the diagnosis of diffuse astrocytic gliomas [25]. MGMT methylation is recommended both for glioblastoma and diffuse hemispheric glioma assessment [25]. Loss of nuclear ATRX expression should prompt additional investigations to exclude diffuse hemispheric glioma, H3.3 G34-mutant (CNS WHO grade 4). All of the tumors were regularly assessed across all centers in our cohort (100%, 81%, and 87.3%, respectively). According to EANO, combined + 7/–10 signature, EGFR amplification, and TERT promoter mutation status should be included in IDH wt diffuse astrocytic gliomas with retained nuclear ATRX expression lacking histological features of WHO grade IV (microvascular proliferation and necrosis) to allow for a diagnosis of IDH wt glioblastoma [6, 21, 25]. However, although without explicitly testing the 3 genetic parameters (TERT promoter mutation, EGFR gene amplification, combined + 7/ − 10 signature) in formerly WHO grade II and III IDH-wildtype astrocytomas which have been deleted in the current WHO classification, the resulting diagnostic failure should be small. A diffuse and astrocytic IDH-wildtype tumor without microvascular proliferation and/or necrosis and without one of the 3 molecular glioblastoma defining markers mentioned above and after excluding diffuse hemispheric or midline gliomas (with also poor clinical prognosis) would be strictly spoken unclassifiable according to the current WHO classification 2021 [16]. Clearly, comprehensive additional workup of these rare cases would be performed and sometimes a surprising diagnosis may evolve. Nevertheless, these cases will be very rare and should not significantly influence the results of our study.

The WHO grade was not associated with PFS and OS between WHO grades II and III. Mean PFS and OS were 12.5 and 27 months, respectively. The OS in our data set is slightly longer than the reported survival of IDH1/2 wt astrocytomas (23.8 months) in a cohort of 67 patients and considerably shorter than the OS of IDH1/2 wt astrocytomas WHO II (59 months) [1, 17, 26, 27]. Regarding PFS and OS IDH1/2 wt astrocytomas, WHO III behave like WHO grade IV tumors [1, 17, 26]. With only 21.5% of all patients in our cohort being WHO grade II and 78.5% WHO grade III, the tendency for a higher recurrence rate and a lower OS reflects the importance and accuracies of the cIMPACT-NOW update to the WHO classification of CNS tumors [1, 5, 6, 16]. Consensus has been reached that EGFR amplification or combined complete + 7/ − 10 chromosome signature or TERT promoter mutation constitute the minimal molecular criteria for identifying an aggressive IDH wt diffuse astrocytic glioma whose clinical course would follow that of an IDH wt grade 4 tumor, despite appearing histologically as a WHO grade II or III [1]. It is thought that these three molecular features are associated with shorter survival and outcomes similar to IDH wt glioblastoma [8, 9, 21, 28–30]. These molecular associations were confirmed in our cohort by a TERT mutation or strong EGFR amplification, which were linked to shortened survival in univariate analysis. However, in a multivariate analysis, only EGFR focal high-level copy number gains remained statistically significant. As TERT promotor mutation analysis was only performed in a subset of patients, small numbers might account for differing results. Concurring with other data, low-level EGFR copy number gains are insufficient to qualify a tumor as EGFR-amplified and did not impact PFS or OS in our cohort [31].

Response to chemotherapy with alkylating substances is significantly better in IDH wt glioblastoma when the MGMT promoter is methylated [32]. Promoter methylation is detected in about 40% of all patients across IDH wt grade II-III tumors and glioblastomas [27, 33]. To date, there is only limited data suggesting a prognostic role of MGMT promoter methylation in IDH 1/2 wt astrocytomas for chemotherapy response and overall survival [27]. The outcomes from the randomized, open-label, phase III CATNON trial in patients with 1p/19q non–co-deleted anaplastic gliomas indicated futility of concurrent temozolomide with radiation and adjuvant temozolomide in patients with IDH 1/2 wildtype tumors. Benefit was restricted to adjuvant treatment in IDH-mutant tumors. [12, 13] A post-hoc analysis from the CATNON study population, identifying 159 IDH 1/2 wt tumors with molecular features of a glioblastoma, similarly revealed no additional benefit of temozolomide in regard to PFS and OS compared to radiotherapy alone [34]. MGMT promoter methylation provided no clinical benefit with either concurrent or adjuvant temozolomide [12, 13]. This observation falls in line with our data adding prove that MGMT methylation status does not have similar prognostic significance for response to therapy and survival as known from glioblastoma.

Some studies showed that older age was associated with earlier tumor recurrence and a shorter OS [35]. As expected, age markedly impacted PFS and OS in our study. We observed that older patients (> 60 years) had a significantly shorter PFS. The cut-off date for older age and increased risk of shortened survival differs between 40 and 64 years of age [17, 24, 36]. Age is also a known and recognized negative prognostic factor in glioblastoma [37]. With age, the burden of comorbidity and frailty also increases. Both proved to be predictors of poor OS in patients with glioblastoma [37–39]. However, frailty is not limited to older age and is thought to be independently associated with a worse prognosis [38]. A rising number of studies confirmed that age per se is a lesser influential factor for survival, but that performance status and frailty are crucial for survival prediction [38, 40]. The impact of frailty on IDH 1/2 wt astrocytoma is largely unknown, and it remains to be seen whether lessons learned from glioblastoma can be extrapolated to other entities [39].

The role of surgical resection in IDH wt astrocytomas is still a matter of debate and has not been addressed in larger studies. We observed a significant difference between PFS after GTR, STR, and biopsy. The extent of resection indicated that approximately 40% of patients had a GTR independent of the treating center. Patients who underwent biopsy alone had a significantly lower PFS, which was associated with markedly reduced OS rates. Most studies agree that GTR generates much lower tumor recurrence rates than STR [41–44]. This supports primary surgical treatment recommendations in terms of GTR. This analysis substantiates the importance of surgical resection improving time to recurrence and overall survival independent of WHO grade, molecular markers, age, or other factors. However, GTR was not superior to STR in terms of PFS and OS. In accordance with our findings, a systematic review and meta-analysis of > 12,000 patients concluded that the only factor increasing PFS in elderly patients with high-grade gliomas was a GTR [45].

Unlike the extent of resection, the decision for adjuvant treatment did not differ across the six participating centers. Although treatment regimens for glioblastoma have been homogenized, there are no clear treatment guidelines for IDH wt diffuse astrocytomas with molecular features of glioblastoma [25]. This underlines the requirement for more clinical trials to generate more comprehensive evidence. Although a clear advantage of concurrent radio-chemotherapy in terms of PFS and OS is missing in this cohort, the data suggests that treatment according to Stupp protocol should be considered as a valuable option for patients with IDH wt astrocytoma.

## Limitations of this study

Although this is the largest series of IDH1/2 wt grade II and III gliomas, our study harbors drawbacks. First, because of the inherent retrospective design, it was impossible to control for treatment regimens after surgery that might affect progression-free and overall survival. Because the original clinical diagnoses were included without central neuropathological review, the data was not homogenized for specific diagnostic algorithms but instead represent the clinical interpretation of current EANO and WHO diagnostic criteria. Finally, because our cohort represents the combined data from 6 different medical centers, treatment decisions based on local practice may influence outcome data.

## Conclusion

This study showed that the clinical course of patients with WHO II and III IDH1/2 wt astrocytomas is similar. No differences in PFS for WHO grade II and III IDH1/2 wt astrocytomas was evident under the same treatment regimens. Persistent nuclear ATRX expression and high EGFR amplification were associated with a worse prognosis, whereas MGMT methylation status did not affect treatment response and survival. The main prognostic factors were the surgical resection, age, and presence of EGFR amplification.

### Supplementary Information

Below is the link to the electronic supplementary material.

ESM 1 (PNG 183 kb)

High resolution image (TIF 4690 KB)

Supplementary file2 (DOCX 14 KB)

## Data Availability

All datasets analyzed in this study are available from the corresponding author on reasonable request.
