# Supplementary figures and images for: Treatment outcome of IDH1/2 wildtype CNS WHO grade 4 glioma histologically diagnosed as WHO grade II or III astrocytomas
Source: J Neurooncol. 2024 Feb 7;167(1):133–44. doi: 10.1007/s11060-024-04585-7 (PMC10978634; doi:10.1007/s11060-024-04585-7)

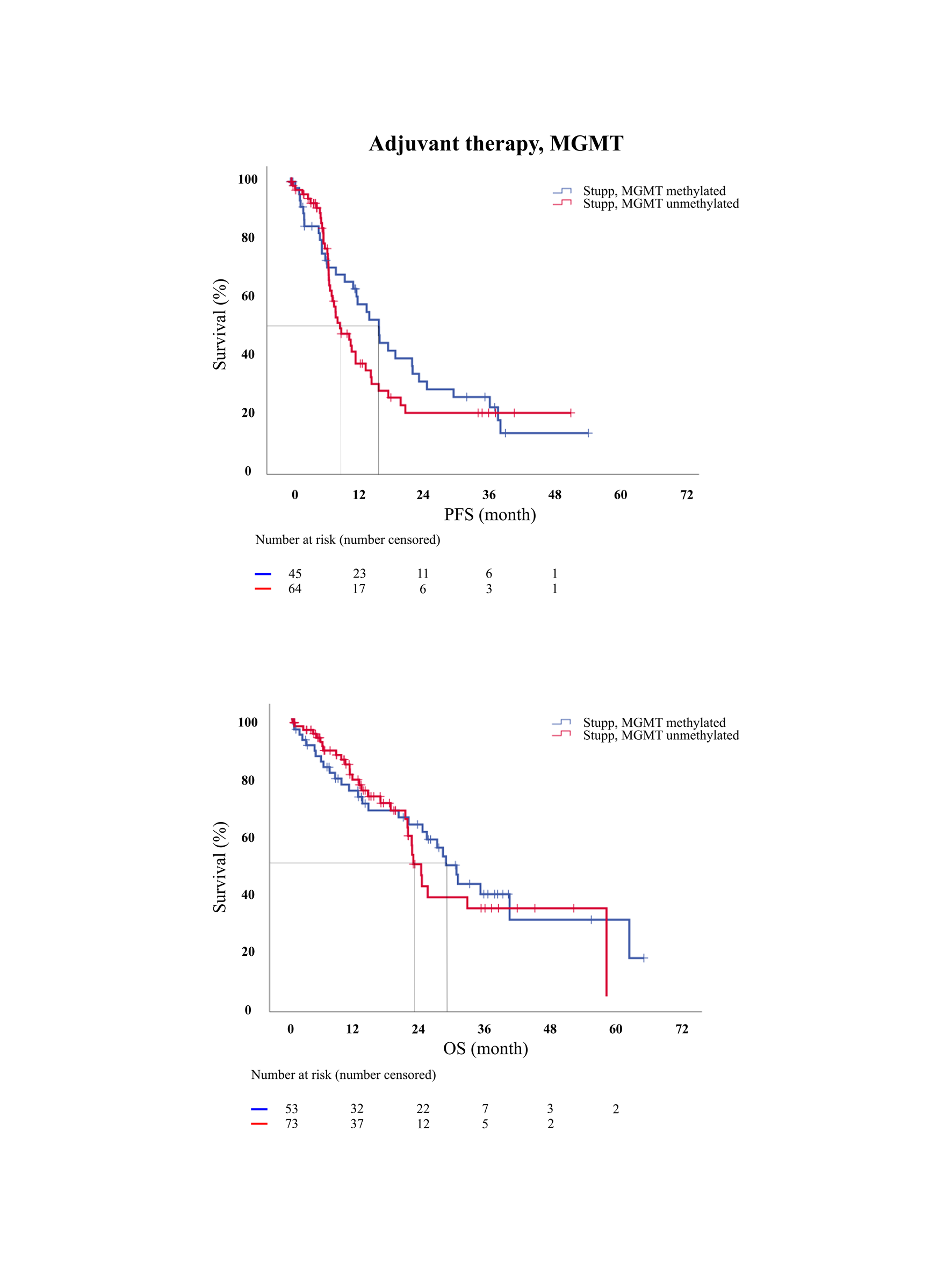

Supplement: Supplementary file 1 — ESM 1 (PNG 183 kb) [file 11060_2024_4585_Fig3_ESM.png]
