# Supplementary material for: Treatment outcome of IDH1/2 wildtype CNS WHO grade 4 glioma histologically diagnosed as WHO grade II or III astrocytomas
Source: J Neurooncol. 2024 Feb 7;167(1):133–44. doi: 10.1007/s11060-024-04585-7 (PMC10978634; doi:10.1007/s11060-024-04585-7)
Supplement: Supplementary file 3 — Supplementary file2 (DOCX 14 KB) [file 11060_2024_4585_MOESM2_ESM.docx]

Table 3. Patient characteristics and treatment regimens in participating neurosurgical centers

|  | **Neurosurgical Center** | | | | | |
| --- | --- | --- | --- | --- | --- | --- |
|  | **1** | **2** | **3** | **4** | **5** | **6** |
| **Patients**, n | 30 | 8 | 27 | 34 | 27 | 31 |
| **Age**, mean (SD) | 57 (13) | 57 (11) | 52 (15) | 64 (14) | 59 (13) | 59 (13) |
| **ECOG** on admission, mean (SD) | 0.5 (0.7) | 2.0 (0.8) | 0.6 (0.7) | 1.3 (0.9) | 0.5 (0.6) | 1.2 (1.0) |
| **GTR**, n (%) | 7 (23) | 0 | 23 (85) | 16 (47) | 5 (19) | 8 (26) |
| **Adjuvant treatment** (Stupp), n (%) | 24 (80) | 8 (100) | 21 (78) | 18 (53) | 18 (72) | 22 (71) |
| **PFS**, months (SE) | 16.6 (2.9) | 13.8 (4.8) | 22.7 (3.5) | 21.8 (3.9) | 20.6 (4.0) | 9.8 (1.0) |
| **OS**, months (SE) | 26.7 (3.4) | 24.7 (4.7) | 40.1 (1.7) | 26.1 (4.2) | 21.6 (3.0) | 35.5 (5.7) |
